# Emergency department and inpatient utilization among U.S. older adults with multiple chronic conditions: a post-reform update

**DOI:** 10.1186/s12913-020-4902-7

**Published:** 2020-02-03

**Authors:** SangNam Ahn, Mustafa Hussein, Asos Mahmood, Matthew Lee Smith

**Affiliations:** 1 The Division of Health Systems Management and Policy, School of Public Health, The University of Memphis, Memphis, 133 Robison Hall, Memphis, TN 38152 USA; 2 Center for Population Health and Aging, Texas A&M University, College Station, 212 Adriance Lab Rd, 1266 TAMU, College Station, TX 77843 USA; 3 Joseph J. Zilber School of Public Health, University of Wisconsin, Milwaukee, WI 53205 USA; 4 Institute for Research on Poverty, University of Wisconsin, Madison, WI 53706 USA; 5 College of Public Health, The University of Georgia, 30602 Athens, GA USA

**Keywords:** Affordable care act, Multiple chronic conditions, Emergency department (ED) visits, Inpatient visits, Length of stay (LOS), Older adults, Medicare

## Abstract

**Background:**

The Affordable Care Act (ACA) was enacted to enhance access to care primarily among nonelderly and low-income populations; however, several provisions addressed key determinants of emergency department (ED) and inpatient visits among Medicare beneficiaries over age 65 years. We take stock of the overall changes in these visits among older Medicare beneficiaries, focusing on those with multiple chronic conditions (MCCs), and provide a nationally representative post-reform update.

**Methods:**

We analyzed a sample of 32,919 older adults (65+) on Medicare from the 2006–2015 Medical Expenditure Panel Survey (MEPS). Using a survey-weighted two-part model, we examined changes in ED visits, inpatient visits, and length of stay (LOS) by MCC status, before (2006–2010), during (2011–2013), and after the ACA (2014–2015).

**Results:**

Prior to the ACA, 18.1% of Medicare older adults had ≥1 ED visit, whereas 17.1% had ≥1 inpatient visits, with an average of 5.1 nights/visit. Following ACA reforms, among those with 2+ chronic conditions, the rate of ever having an ED visit *increased* by 4.3 percentage points [95% confidence intervals [CI]: 2.5, 6.1, *p* < 0.01], whereas the rate of inpatient visits decreased by 1.4 percentage points [95%CI: − 2.9, 0.2, *p* < 0.1], after multivariable adjustment.

**Conclusions:**

We found sizable increases in ED visits and nontrivial decreases in inpatient visits among older Medicare beneficiaries with MCCs, underscoring the continuing need for improving access to and quality of care among older adults with MCCs to decrease reliance on the ED and reduce preventable hospitalizations.

## Background

Having multiple chronic conditions (MCCs), the coexistence of two or more chronic conditions [1], has emerged as a serious public health concern among older adults in the United States [2]. More than 8 out of 10 older adults suffered from MCCs in 2014 [3], requiring ongoing disease management over a period of years or decades. MCCs are associated with decreased quality of life and functional decline among older adults [1, 4]. Compared to older adults without a chronic condition, older adults with one, two, and three or more conditions are likely to experience a loss of 4.7, 7.9, and 10.8 quality-adjusted life years (QALY), respectively [5]. The presence of MCCs increases the risk of developing functional limitation [6]; onset of moderate functional limitation is far more likely among 80-year-olds with MCCs than their counterparts without MCCs (50% vs. 22%, respectively) [7].

Further, MCCs are associated with a significant financial burden due to increasing ambulatory, emergency department, and hospital visits [8]. In the Medicare program, the annual Medicare payments for a beneficiary grew from $7172, to $14,931, to $32,498 when the beneficiary had one, two, and three or more chronic conditions, respectively [9]. As the population ages and Baby Boomers continue to retire on to Medicare, the impacts of MCCs on Medicare spending, especially Medicare Part A whose funds are expected to be depleted by 2026 [10], warrant urgent scrutiny.

The Patient Protection and Affordable Care Act (ACA) of 2010 was enacted to accomplish the Triple Aim: better healthcare, better health outcomes, and better value [11–13]. Although ACA’s coverage provisions were primarily focused on the non-elderly population [14], the law had several key provisions that specifically applied to older adults in Medicare. First and foremost, the law took direct aim at reducing Medicare spending growth through the Independent Payment Advisory Board as well as various provisions related to healthcare quality, utilization, and payment [10, 15]. As of January 2011, the ACA eliminated cost-sharing for preventive services and authorized coverage of personalized prevention plans, including Annual Wellness Visits under Part B [16]. Although fee-for-service payment models still dominate the healthcare system [17], older Medicare beneficiaries may have also benefitted from the growth in innovative payment and delivery models (e.g., accountable care organizations, bundled payment, and patient-centered medical homes) [18–20]. Further, ACA’s strict regulations and penalties related to hospital utilization (e.g., emergency department [ED] visits, inpatient visits, and length of stay [LOS]), including the Hospital Readmission Reduction Program (HRRP) [21], may have also affected older Medicare beneficiaries, especially those with MCCs. Table 1 lists these and other key ACA provisions relevant to older adults with MCCs.

**Table 1:** Key Affordable Care Act (ACA) Provisions Relevant to Older Adults with Multiple Chronic Conditions

Key Provisions	Effective Implementation Date
Quality	
Improve care coordination for dual eligibles by creating a new office within the CMS services.	March 2010
Reduce Medicare payments to certain hospitals for hospital-acquired conditions by 1%.	October 2015
Reduce Medicare payments that would otherwise be made to hospitals by specified percentages to account for excess (preventable) hospital readmissions.	October 2012
Provide incentives to Medicare and Medicaid beneficiaries to complete behavior modification programs.	January 2011
Access	
Provide payments totaling $400 million in fiscal years 2011 and 2012 to qualifying hospitals in counties with the lowest quartile Medicare spending.	January 2011
Provide a 10% bonus payment to primary care physicians in Medicare from 2011 through 2015.	January 2011
Reduce Medicare Disproportionate Share Hospital (DSH) payments initially by 75% and increase payments.	October 2014
Eliminate cost-sharing for Medicare covered preventive services and waive the Medicare deductible for colorectal cancer screening tests.	January 2011
Authorize Medicare coverage of annual personalized prevention plan services.	January 2011
Cost	
Increase the Medicare Part A (hospital insurance) tax rate on wages by 0.9% (from 1.45 to 2.35%) on earnings over $200,000 for individual taxpayers and $250,000 for married couples.	January 2013
Restructure payments to Medicare Advantage (MA) plans by setting payments to different percentages of Medicare fee-for-service (FFS) rates.	January 2011
Establish an Independent Payment Advisory Board to submit legislative proposals containing recommendations to reduce the per capita rate of growth in Medicare spending.	April 2013
Cost & Quality (Alternative Payment Models)	
Allow providers organized as accountable care organizations (ACOs) that voluntarily meet quality thresholds to share in the cost savings they achieve for the Medicare program.	January 2012
Establish a hospital value-based purchasing program in Medicare to pay hospitals based on performance on quality measures.	October 2012
Establish a national Medicare pilot program to develop and evaluate paying a bundled payment.	January 2013
[Promote] patient-centered medical home models for high-need applicable individuals.	January 2011

Rather than evaluating the specific effects of each individual provision on older adults with MCCs [22–25], which took effect between 2011 and 2014, the present study aims to examine overall changes in ED visits, inpatient visits, and length of hospital stays among older Medicare beneficiaries with MCCs before (2006–2010), during (2011–2013), and after the ACA (2014–2015). By providing a post-reform update with nationally representative estimates, this analysis can inform continuing efforts to improve care quality and reduce spending among older Medicare beneficiaries with MCCs in this era with a precarious healthcare future.

## Methods

### Sample and data

We analyzed data from the Medical Expenditure Panel Survey (2006–2015), a nationally representative survey of the civilian non-institutionalized population. We had an eligible sample of 34,721 MEPS respondents who received Medicare and were age 65 years or older. The vast majority of our study covariates, including outcomes, main demographics, and chronic conditions, were virtually fully available for the entire sample (0 to < 0.5% missing). Data for only 4 key variables (education, self-reported general and mental health status, and having a usual source of care) had missing for < 1.5% of the eligible sample (Additional file 1: Table S1). With such low missing data rates, our final analytical sample included all respondents with complete data for all study covariates (*n* = 32,919). Excluded respondents (only 5% of the eligible sample) had greater ED and inpatient utilization, were more likely to have had a myocardial infarction or a stroke (and activity limitations), but had fewer chronic conditions overall. On average, excluded respondents were older, poorer, less likely to be white, and less likely to be married (Additional file 1: Table S2). Given the small size and worse characteristics of excluded participants, we did not expect their exclusion to materially bias our findings; if anything, our estimates might be slightly conservative.

We linked respondents’ data in MEPS annual files to their respective records from the Medical Conditions files, and then pooled linked datasets for years 2006–2015. Our data cover three distinct periods with respect to the ACA: pre-ACA (2006–2010), implementation period of ACA provisions relevant to older adults with MCCs (2011–2013), and post-ACA (2014–2015).

### Measures

#### Outcomes

As primary outcomes, we first documented the prevalence of having any (at least one) emergency department (ED) visit, hospital inpatient visit, and overnight inpatient stay. As secondary outcomes, we analyzed counts of ED visits, inpatient visits, and LOS (total and average).

#### Chronic conditions

We identified chronic conditions by using the definitions developed by Hwang and colleagues, and adopted by the Agency for Healthcare Research and Quality [26, 27], applied to International Classification of Diseases 9th Revision (ICD-9) 3-digit codes in the MEPS Medical Conditions files. We then calculated the total number of unique chronic conditions for each respondent, and categorized them as having 0, 1, 2, 3, 4, or 5+ chronic conditions. Those with ≥2 conditions were classified as having MCCs.

#### Covariates

Our analysis used data about respondents’ characteristics known to be related to ED visits, inpatient services, and having MCCs. Respondent sociodemographic characteristics included age, gender, race/ethnicity, language, marital status, Census region, income relative to the federal poverty line (FPL), and education. To measure respondents’ health status, we included self-rated general and mental health, activity limitations (physical and cognitive), and their chronic condition(s) (e.g., high blood pressure, diabetes, heart disease, stroke, and asthma). We also considered respondents’ access to care including types of payer (i.e., Medicaid, private insurance), having a usual source of care, receiving needed medical care, and getting needed prescription drugs. These factors are key determinants of ED use and hospitalization. Detailed levels of these covariates are reported in Table 2.

**Table 2:** Sample Characteristics Before and After the Affordable Care Act (ACA), MEPS 2006–2015

	*Pre-ACA* (2006–2010)	*ACA* (2011–2013)	*Post-ACA* (2014–2015)	Overall
Sample Size	15,548	10,313	7058	32,919
Population Represented	16,723,986	11,515,758	8,256,622	36,496,366
Outcomes				
Any ED Visits	18.1	18.2	21.0	18.8
Number of ED Visits (if 1+), Mean (SD)	1.4 (0.9)	1.4 (0.8)	1.6 (1.1)	1.4 (0.9)
Any Inpatient Visits	17.1	15.2	15.6	16.2
Number of Inpatient Visits (if 1+), Mean (SD)	1.4 (0.8)	1.4 (0.8)	1.5 (1.0)	1.4 (0.9)
Total Inpatient Nights (if 1+), Mean (SD)	7.6 (10.9)	7.4 (13.8)	7.8 (11.6)	7.6 (12.0)
Average Number of Nights/Inpatient Visit (if 1+), Mean (SD)	5.1 (6.5)	5.0 (7.5)	4.9 (6.2)	5.0 (6.8)
Demographics				
Age, Mean (SD)	74.1 (6.3)	73.7 (6.4)	73.5 (6.3)	73.9 (6.3)
Female (%)	57.4	55.7	55.9	56.5
Race/Ethnicity (%)				
White (Non-Hispanic)	80.8	79.5	77.4	79.6
Black (Non-Hispanic)	7.9	8.3	8.6	8.2
Other (Non-Hispanic)	4.5	5.1	6.3	5.1
Hispanic	6.8	7	7.7	7.1
Poverty Level (%)				
Poor/Negative-Income (< 100%FPL)	9.3	9.1	9.2	9.2
Near-Poor (100–125%FPL)	7.4	6.2	6.1	6.7
Low-Income (125–200%FPL)	18	18	16.3	17.6
Middle-Income (200–400%FPL)	29.9	29.3	27.2	29.1
High-Income (≥400%FPL)	35.4	37.4	41.3	37.4
Education (%)				
Less than High School	23.7	18.4	16.6	20.5
High School Diploma	35.3	32.4	30.5	33.3
Some College	18.6	22.9	24.4	21.3
Bachelor’s Degree or Higher	22.3	26.3	28.6	25.0
Census Region (%)				
Northeast	19.9	18.7	18.7	19.2
Midwest	22.1	22.8	22.3	22.4
South	37.0	36.8	37.2	37.0
West	21.0	21.7	21.8	21.4
Marital Status (%)				
Single/Never Married	3.5	4.2	4.2	3.9
Widowed/Divorced/Separated	42.3	41.4	40	41.5
Married/Cohabiting	54.2	54.5	55.8	54.6
Interview Not In English (%)	3.3	4.2	4.6	3.9
Health Status (%)				
General Health Status (Self-Rated)				
Excellent	14.9	17.1	17.6	16.2
Very Good	29.9	30.5	31.1	30.4
Good	32.8	32.5	31.4	32.4
Fair	16.2	15.0	14.6	15.5
Poor	6.2	4.9	5.3	5.6
Mental Health Status (Self-Rated)				
Excellent	27.8	29.5	30.2	28.9
Very Good	30.9	30.4	29.1	30.3
Good	30.8	30.1	29.8	30.3
Fair	8.2	7.9	8.5	8.1
Poor	2.4	2.3	2.5	2.4
Any Physical or Cognitive Limitations (Self-Reported)	59.2	56.6	57.4	58.0
Chronic Conditions (%)				
Number of Chronic Conditions				
0	7.4	7.2	8.2	7.5
1	12.6	11.1	11.4	11.8
2	16.7	16.4	16.4	16.5
3	16.5	16.9	16.7	16.7
4	14.7	15.2	15.8	15.1
5+	32.2	33.2	31.6	32.4
High Blood Pressure	67.8	69.5	68.2	68.4
Coronary Heart Disease	19.3	19.9	18.9	19.4
Angina	9.0	7.7	7.1	8.2
Myocardial Infarction	12.3	11.8	12.4	12.2
Other Heart Disease	25.0	27.1	29.3	26.6
Stroke	12.2	12.2	11.6	12.0
Diabetes	20.6	21.4	23.2	21.4
Emphysema	6.5	6.5	6.2	6.4
Asthma	8.8	8.7	9.0	8.8
Arthritis	58.1	59.4	60.2	59.0
Insurance and Access to Care (%)				
Had Any Private Insurance	52.3	52.7	54.6	53.0
Had Any Public Insurance	47.7	47.3	45.4	47.0
Had Medicaid (Ever)	9.7	10.3	10.0	10.0
Have a Usual Source of Care	93.9	93.9	94.0	93.9
Cannot Get Needed Medical Care	0.9	1.1	1.6	1.1
Cannot Get Needed Prescription Drugs	1.4	1.7	2.2	1.7

### Statistical analysis

The goal of our analysis was to provide an update of where levels of ED visits and inpatient stays stand among older adults with MCCs following relevant ACA reforms, relative to the pre-ACA period. In our statistical models, this was accomplished by interacting a period indicator (pre-ACA = 0, post-ACA = 1) with chronic condition categories (having 5+, 4, 3, 2, 1, vs. 0), while including the main effects of these variables as well as the aforementioned confounding covariates. Since we are interested in the specific associations of having MCCs with ED/inpatient utilization, we adjusted for potential confounding by the following sets of covariates: 1) sociodemographic factors, which predispose (e.g., age) or enable (e.g., income) utilization; 2) particular conditions respondents had (e.g. stroke, myocardial infarction, asthma), which drive both the burden (count) of chronic conditions and the need for ED/inpatient utilization; and 3) additional insurance (Medicaid or private) and access-related factors (e.g., having a usual source of care), which also enable or create the need for ED/inpatient utilization. Our preferred model specification fully adjusts for these three sets of potential confounders. Additionally, we assessed the changes in model fit as we sequentially adjusted for these covariate sets.

We analyzed binary outcomes (prevalence of having ≥1 utilization event [i.e., visit or night]) in logit models. For count outcomes, we used a two-part, logit-negative binomial model, In the two-part model (known as a hurdle model for count data), a logit model is fitted for the probability of having ≥1 utilization event, and concurrently a negative binomial regression model is fitted for the actual count of events, conditional on a positive utilization event. By doing so, this two-part model handles the severely right-skewed nature of count distributions, with a concentrated mass of zeros on the left-hand side of the distribution and a very long right tail [28]. Two-part models also allow recovering population-average estimates of change in outcome levels from the entire sample, as opposed to conditional estimates obtained from models fit only to the subsample with ≥1 event [29]. After estimating each of our logit and two-part models, we recovered the adjusted, average marginal probability (of having ≥1 event) and count of events, by ACA period and MCC category. Finally, we estimated the pre-post-ACA changes in probabilities and counts for each MCC category.

For our logit models of binary outcomes, we assessed the goodness of fit using a modified version of the Hosmer-Lemeshow test for complex survey data [30]. *P*-values for our preferred fully adjusted models were all between 0.3 and 0.4, indicating adequate fit. For the hurdle models of count data, we used Akaike and Bayesian Information Criteria (AIC & BIC) to compare model specifications. Our fully-adjusted models had the smallest AIC and BIC, indicating best fit among all tested specifications.

All models were estimated using maximum likelihood estimation. All estimates were also generated using Stata’s “*svy*” prefix, which uses survey weights to make estimates nationally representative. This prefix also calculates linearized standard errors, which account for MEPS’s complex, multi-stage sampling. All analyses were performed in Stata 14.2 (StataCorp, College Station, TX).

## Results

As Table 2 shows, respondents were 74 years-old on average (±6.3 years), and 56.5% were female, 79.6% were non-Hispanic White, and 33.5% were poor or low-income (< 200% FPL). Fifty-eight percent had physical or cognitive limitations, while 80.6% had MCCs, including 32.4% with ≥5 chronic conditions. High blood pressure (68.4%) and arthritis (59.0%) were the most prevalent conditions. Besides Medicare, 53% also had private supplementary insurance and 10% received Medicaid. The vast majority (93.9%) had a usual source of care and reported no problems accessing needed care or prescription drugs (97.2%). Sample characteristics were generally stable over the study period.

**Fig. 1:**
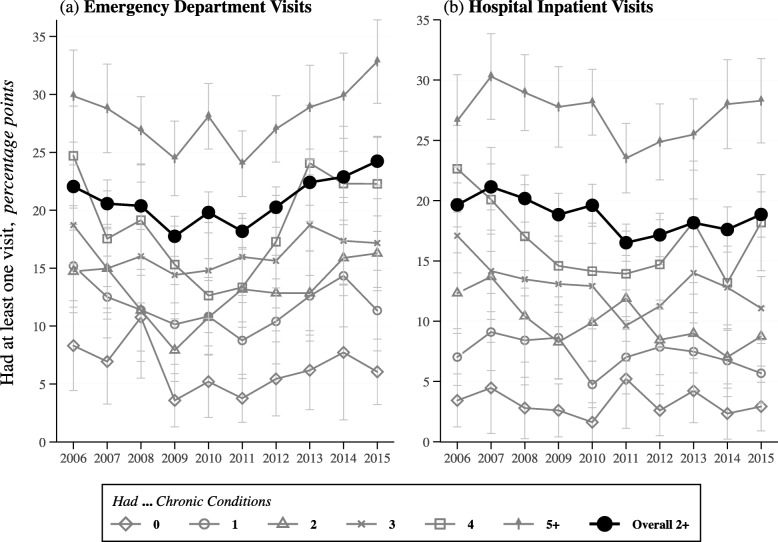
Observed Trends in Emergency Department and Inpatient Visit Probabilities by Multiple Chronic Condition Levels

The rates of having any ED visits and inpatient stays in our sample changed from 18.1 and 17.1% pre-ACA (2006–2010) to 21.0 and 15.6% post-ACA (2014–15), respectively. Among those who ever had a visit, there was an average of 1.4 ED visits and 1.4 inpatient visits, with 5.1 nights spent in the hospital on average per visit, prior to the ACA (Table 2). While there was generally a downward trend in ED visits until 2011 (Fig. 1a), rates increased between 2011 and 2013 among all groups (see Additional file 1: Tables S3-S5 for detailed statistics). The data further reveal some tapering off into 2015, except among those with ≥5 conditions who continued to see a potentially steeper increase. Inpatient visit rates (Fig. 1b) also trended down from 2006 to 2015 for groups with < 4 conditions. However, among those with ≥4 conditions, inpatient visit rates increased after 2011 into 2015.

**Fig. 2:**
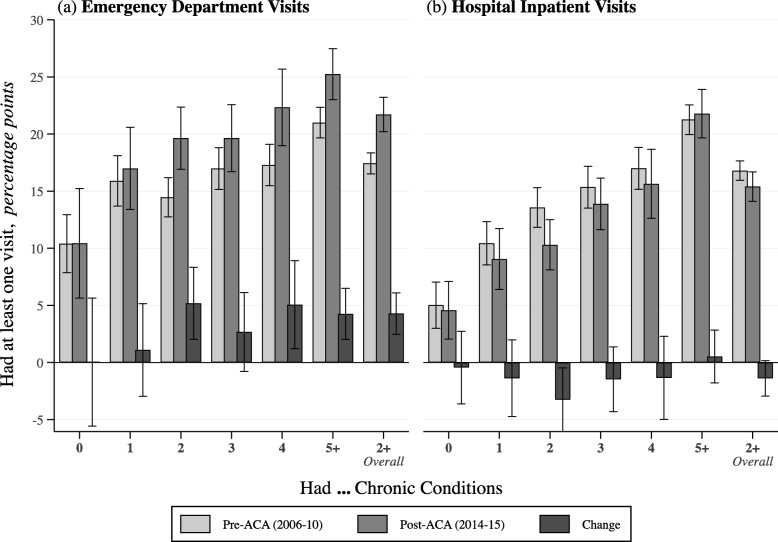
Adjusted Changes in ED and Inpatient Visit Probabilitiesy by Multiple Chronic Condition Levels

**Fig. 3:**
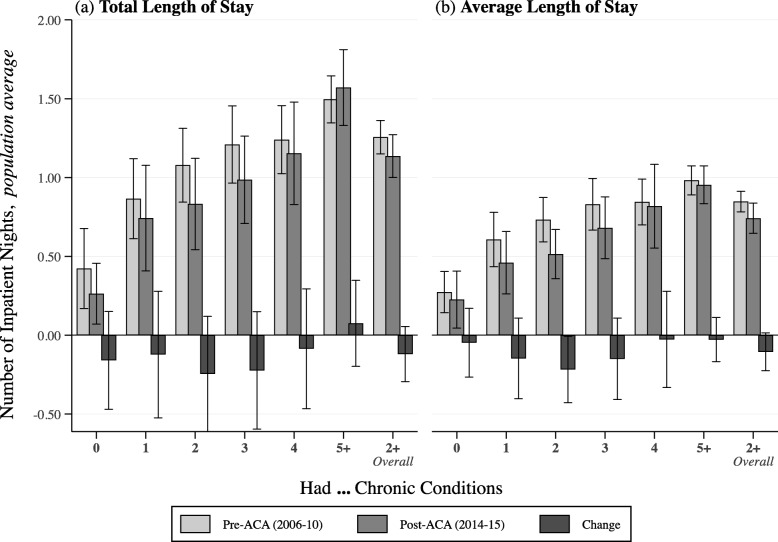
Adjusted Changes in Total Annual Inpatient Nights (Total Length of Stay) and Average Number of Nights per Inpatient Visit (Average Length of Stay) by Multiple Chronic Condition Levels

After multivariable adjustment, ED visit rates (having ≥1 visits) in 2014–2015 were overall higher than the pre-ACA period among all beneficiaries with ≥2 conditions by 4.3 percentage points (95% confidence interval [CI]: 2.5, 6.1) (Fig. 2a). For inpatient visits, we detected a drop in rates among all beneficiaries, especially those with just 2 chronic conditions who experienced a decrease in the probability of having at least 1 visit by 3.3 percentage points (95%CI: − 6.1, − 0.5) (Fig. 2b). Overall, those with ≥2 conditions saw a marginal drop by 1.4 percentage points (95%CI: − 2.9, 0.2) in inpatient visit rates in 2014–2015. Changes in LOS (inpatient nights) also followed a similar pattern (Fig. 3). Finally, we assessed the sensitivity of our findings by dropping education, self-rated general and mental health status, and having a usual source of care from our models. Dropping these four confounders serves to re-include most of the excluded participants from the eligible sample. The sensitivity analyses were less conservative than the main findings because they did not adjust for the confounders (Additional file 1: Table S6).

## Discussion

In the first 2 years following the ACA (2014–2015), we detected sizable increases in ED use and nontrivial decreases in inpatient visits among older Medicare beneficiaries with MCCs. To our knowledge, this is the first study to take a big-picture view and document the overall changes in hospital utilization by MCC status among older Medicare beneficiaries in the context of recent healthcare reform using a large, nationally representative dataset. The ACA was designed to primarily improve access to care among nonelderly and low-income populations, and has been associated with a reduction in the total number of the uninsured from 18.2% in 2010 to 10.4% in 2016 [31]. The reduction in the number of the uninsured was primarily centered on younger age groups: adults ages 19–34 by 42% (8.7 million), 35–54 by 33% (5.6 million), and 55–64 by 33% (2.0 million) [32]. Simultaneously, the total number of Medicare beneficiaries increased by 13.5% from 48.9 million in 2011 to 55.5 million in 2015 [31]. However, there is little evidence about how hospital utilization among older Medicare beneficiaries with MCCs has changed since the ACA was introduced.

Our analysis shows that over time, inpatient visits showed a nontrivial decrease among older Medicare beneficiaries. Such decrease is consistent with existing evidence showing decreasing expenditure on hospital inpatient stays by 6.6%, from 37.8 million in 2005 to 35.4 million in 2014 [33]. There are a couple of plausible explanations for these decreases. First, inpatient visits may have decreased because of the ACA’s enhanced coverage of preventive services under Medicare Part B [34], which eliminated patients’ cost-sharing and introduced free Annual Wellness Visits [24]. The second probable reason would be the introduction of the Hospital Readmissions Reduction Program (HRRP), which penalizes hospitals with above-average readmissions for Medicare patients with preventable conditions, including myocardial infarction, pneumonia, and heart failure [35]. The significant penalty (i.e., 3% of Medicare payments) likely prompted hospitals to proactively avert repeated admissions [23] while potentially motivating hospitals to use observation status [36]. Naturally, hospitals spent a great amount of resources to develop and strengthen care coordination [37], transitional care [38], and adopt voluntary value-based reforms [39] to avoid unnecessary readmissions. Further studies are warranted to investigate the associations of enhanced coverage of preventive services and HRRP with decreased inpatient care.

Our second main finding is the increase in the probability of having ≥1 ED visit by 4.3 percentage points in the post-ACA period among older Medicare beneficiaries with 2+ MCCs. This is a sizable increase of ~ 25% from pre-ACA levels (17%). Interestingly, ED visits showed an increasing pattern despite most study participants had a usual source of care (overall, 93.9%), while only a few participants reported problems accessing needed care (1.1%) or prescription drugs (1.7%). Previous studies found that more ED visits were sensitive to health insurance status [40, 41] or having a usual source of care [42]. Other studies found that ED visits were affected by the severity of patients’ illness or comorbidity [43, 44]. Despite this, the demand for ED might remain relatively inelastic, regardless of having urgent health conditions [45] or a usual source of care [46, 47]. Further studies are warranted to investigate how having a usual source of care could affect ED visits among older Medicare beneficiaries with MCCs. We also postulate that hospital responses to the HRRP might offer an explanation for this pattern as well. The previous literature speculated that the HRRP may have encouraged hospitals to “game the system” by holding more patients in ED or admitting them for observation [23]. Observation stays significantly increased after the HRRP went into effect for target conditions, but not for the non-target conditions [48]. Another study confirmed that the top 10% of hospitals with the largest drop in hospital readmission increased the use of observation status by 25% among Medicare patients returning within 30 days [49].

Our study has key strengths, including providing nationally representative estimates over policy-relevant time periods, and using a validated scheme for identifying and counting chronic conditions among older adults. However, one key has limitation deserves comment. While our goal was to document the changes in ED/inpatient use potentially driven by all ACA reforms relevant to older adults with MCCs, our findings do not necessarily have a causal interpretation as exclusively being due to the ACA. This is because of the lack of an appropriate control group that was not exposed to all of the reforms we discussed. While the non-MCC group in our analysis was arguably the least affected by the ACA, the fact that the non-MCC group is much healthier than their MCC counterparts suggests that they are a different population in terms of other important observable and unobservable ways. Additionally, while 2 years following ACA may be insufficient to detect stable post-reform outcome levels, 2015 is the most recently available year of MEPS data for which chronic conditions can be identified using ICD-9 codes.

## Conclusions

Our evaluation permits a better understanding of overall hospital use patterns among older Medicare beneficiaries with MCCs throughout the course of the ACA. We documented an increase in ED visits but a decrease in inpatient utilization among the population following the ACA. This seemingly paradoxical relationship warrants further examination over longer post-ACA periods, as well as identification of the underlying patient- and system-level causes of such change, in order to improve the access to care and quality of care while containing the healthcare cost among older adults.

## Supplementary information

**Additional file 1: Table S1.** Study Covariates with Missing Data. **Table S2.** Characteristics of Excluded vs. Included Respondents. **Table S3**. Adjusted levels and changes in **emergency department visits** among older adults with multiple chronic conditions (2006–2015). **Table S4**. Adjusted levels and changes in **inpatient visits** among older adults with multiple chronic conditions (2006–2015). **Table S5**. Adjusted levels and changes in **inpatient length of stay** among older adults with multiple chronic conditions (2006–2015). **Table S6.** Changes in ED/Inpatient Utilization Around the ACA Estimated in the Main Sample versus in a Full Sample Excluding 4 Key Covariates with Missing Data.

## Data Availability

The Medical Expenditure Panel Survey dataset are publicly available in the Agency for Healthcare Research and Quality: Medical Expenditure Panel Survey webpage in https://meps.ahrq.gov/mepsweb/data_stats/download_data_files.jsp.

## Abbreviations

ACA: Affordable care act
CI: Confidence intervals
ED: Emergency department
FPL: Federal poverty line.
HRRP: Hospital Readmission Reduction Program
ICD-9: International Classification of Diseases 9th Revision
LOS: Length of stay
MCC: Multiple chronic conditions
MEPS: Medical Expenditure Panel Survey
QALY: Quality-adjusted life years

## References

[1] Boyd CM, Fortin M (2010). Future of multimorbidity research: how should understanding of multimorbidity inform health system design?. Public Health Rev.

[2] Anderson GF (2010). Chronic care: making the case for ongoing care.

[3] Buttorff C, Ruder T, Bauman M (2017). Multiple chronic conditions in the United States. RAND Corporation.

[4] Ryan A, Wallace E, O’Hara P, Smith SM (2015). Multimorbidity and functional decline in community-dwelling adults: a systematic review. Health Qual Life Outcomes.

[5] Jia H, Lubetkin EI (2016). Impact of nine chronic conditions for US adults aged 65 years and older: an application of a hybrid estimator of quality-adjusted life years throughout remainder of lifetime. Qual Life Res.

[6] Lunney JR, Lynn J, Foley DJ, Lipson S, Guralnik JM (2003). Patterns of functional decline at the end of life. JAMA.

[7] Dunlop DD, Manheim LM, Sohn M-W, Liu X, Chang RW (2002). Incidence of functional limitation in older adults: the impact of gender, race, and chronic conditions. Arch Phys Med Rehabil.

[8] McPhail SM (2016). Multimorbidity in chronic disease: impact on health care resources and costs. Risk Manag Healthc Policy.

[9] Schneider KM, O’Donnell BE, Dean D (2009). Prevalence of multiple chronic conditions in the United States’ Medicare population. Health Qual Life Outcomes.

[10] Cubanski J, Neuman T (2016). The Facts on Medicare Spending and Financing. The Henry J. Kaiser Family Foundation.

[11] Berwick DM, Nolan TW, Whittington J (2008). The triple aim: care, health, and cost. Health Aff.

[12] Ahn S, Basu R, Smith ML, Jiang L, Lorig K, Whitelaw N, Ory MG (2013). The impact of chronic disease self-management programs: healthcare savings through a community-based intervention. BMC Public Health.

[13] Blumenthal D, Abrams M, Nuzum R (2015). The affordable care act at 5 years. N Engl J Med.

[14] French MT, Homer J, Gumus G, Hickling L (2016). Key provisions of the patient protection and affordable care act (ACA): a systematic review and presentation of early research findings. Health Serv Res.

[15] The Henry J (2013). Kaiser Family Foundation. Summary of the Affordable Care Act.

[16] Hughes C (2011). What you need to know about the Medicare preventive services expansion. Fam Pract Manag.

[17] Zuvekas SH, Cohen JW (2016). Fee-for-service, while much maligned, remains the dominant payment method for physician visits. Health Aff.

[18] Friedberg MW, Rosenthal MB, Werner RM, Volpp KG, Schneider EC (2015). Effects of a medical home and shared savings intervention on quality and utilization of care. JAMA Intern Med.

[19] Burwell SM (2015). Setting value-based payment goals—HHS efforts to improve US health care. N Engl J Med.

[20] Porter ME, Kaplan RS (2016). How to pay for health care. Harv Bus Rev.

[21] Fonarow GC, Konstam MA, Yancy CW: The hospital readmission reduction program is associated with fewer readmissions, more deaths: time to reconsider. J Am Coll Cardiol. 2017;70(15):1931–4. 10.1016/j.jacc.2017.08.046 28982507

[22] Boozary AS, Manchin J, Wicker RF (2015). The Medicare hospital readmissions reduction program: time for reform. JAMA.

[23] Joynt KE, Jha AK (2013). A path forward on Medicare readmissions. N Engl J Med.

[24] Jensen GA, Salloum RG, Hu J, Ferdows NB, Tarraf W (2015). A slow start: use of preventive services among seniors following the affordable care Act's enhancement of Medicare benefits in the US. Prev Med.

[25] Dusetzina SB, Keating NL (2016). Mind the gap: why closing the doughnut hole is insufficient for increasing Medicare beneficiary access to oral chemotherapy. J Clin Oncol.

[26] Paez KA, Zhao L, Hwang W (2009). Rising out-of-pocket spending for chronic conditions: a ten-year trend. Health Aff.

[27] Gerteis J, Izrael D, Deitz D, LeRoy L, Ricciardi R, Miller T, Basu J, Rockville MD (2014). Multiple Chronic Conditions Chartbook: 2010 Medical Expenditure Panel Survey Data. Edited by Agency for Health Research and Quality.

[28] Long JS, Freese J (2006). Regression analysis for categorical dependent variables using Stata.

[29] Belotti F, Deb P, Manning WG, Norton EC (2015). Twopm: two-part models. Stata J.

[30] Archer KJ, Lemeshow S (2006). Goodness-of-fit test for a logistic regression model fitted using survey sample data. Stata J.

[31] The HJ (2017). Kaiser Family Foundation. Key Facts about the Uninsured Population.

[32] Garrett AB, Gangopadhyaya A (2016). Who gained health insurance coverage under the ACA, and where do they live?.

[33] McDermott KW, Elixhauser A, Sun R, Rockville MD (2017). Trends in Hospital Inpatient Stays in the United States, 2005–2014. HCUP Statistical Brief #225. Edited by Agency for Healthcare Research and Quality.

[34] Koh HK, Sebelius KG (2010). Promoting prevention through the affordable care act. N Engl J Med.

[35] McIlvennan CK, Eapen ZJ, Allen LA (2015). Hospital readmissions reduction program. Circulation.

[36] Albritton J, Belnap TW, Savitz LA (2018). The effect of The hospital readmissions reduction program on readmission and observation stay rates for heart failure. Health Aff.

[37] Peikes D, Chen A, Schore J, Brown R (2009). Effects of care coordination on hospitalization, quality of care, and health care expenditures among Medicare beneficiaries: 15 randomized trials. JAMA.

[38] Verhaegh KJ, MacNeil-Vroomen JL, Eslami S, Geerlings SE, de Rooij SE, Buurman BM (2014). Transitional care interventions prevent hospital readmissions for adults with chronic illnesses. Health Aff.

[39] Ryan AM, Krinsky S, Adler-Milstein J, Damberg CL, Maurer KA, Hollingsworth JM (2017). Association between hospitals’ engagement in value-based reforms and readmission reduction in the hospital readmission reduction program. JAMA Intern Med.

[40] Miller S (2012). The effect of insurance on emergency room visits: an analysis of the 2006 Massachusetts health reform. J Public Econ.

[41] Gindi RM, Cohen RA, Kirzinger WK. Emergency room use among adults aged 18–64: Early release of estimates from the National Health Interview Survey, January–June 2011. National Center for Health Statistics: Atlanta, GA. May 2012. Available from: http://www.cdc.gov/nchs/nhis/releases.htm.

[42] Weber EJ, Showstack JA, Hunt KA, Colby DC, Callaham ML (2005). Does lack of a usual source of care or health insurance increase the likelihood of an emergency department visit? Results of a national population-based study. Ann Emerg Med.

[43] Wagner TH, Guendelman S (2000). Healthcare utilization among Hispanics: findings from the 1994 minority health survey. Am J Manag Care.

[44] Cunningham PJ, Clancy CM, Cohen JW, Wilets M (1995). The use of hospital emergency departments for nonurgent health problems: a national perspective. Med Care Res Rev.

[45] Taubman SL, Allen HL, Wright BJ, Baicker K, Finkelstein AN (2014). Medicaid increases emergency-department use: evidence from Oregon's health insurance experiment. Science.

[46] Rust G, Fryer GE, Phillips RL, Daniels E, Strothers H, Satcher D (2004). Modifiable determinants of healthcare utilization within the African-American population. J Natl Med Assoc.

[47] Rust G, Ye J, Baltrus P, Daniels E, Adesunloye B, Fryer GE (2008). Practical barriers to timely primary care access: impact on adult use of emergency department services. Arch Intern Med.

[48] Zuckerman RB, Sheingold SH, Orav EJ, Ruhter J, Epstein AM (2016). Readmissions, observation, and the hospital readmissions reduction program. N Engl J Med.

[49] Noel-Miller C, Lind K. Is Observation Status Substituting For Hospital Readmission?. Health Affairs Blog, October 28, 2015. 10.1377/hblog20151028.051459.

