# Supplementary material for: Emergency department and inpatient utilization among U.S. older adults with multiple chronic conditions: a post-reform update
Source: BMC Health Serv Res. 2020 Feb 3;20:77. doi: 10.1186/s12913-020-4902-7 (PMC6998236; doi:10.1186/s12913-020-4902-7)
Supplement: Supplementary file 1 — Additional file 1: Table S1. Study Covariates with Missing Data. Table S2. Characteristics of Excluded vs. Included Respondents. Table S3. Adjusted levels and changes in emergency department visits among older adults with multiple chronic conditions (2006–2015). Table S4. Adjusted levels and changes in inpatient visits among older adults with multiple chronic conditions (2006–2015). Table S5. Adjusted levels and changes in inpatient length of stay among older adults with multiple chronic conditions (2006–2015). Table S6. Changes in ED/Inpatient Utilization Around the ACA Estimated in the Main Sample versus in a Full Sample Excluding 4 Key Covariates with Missing Data. [file 12913_2020_4902_MOESM1_ESM.docx]

**Supplementary Table 1.** Study Covariates with Missing Data.

|  | ***n***  **Missing** | ***n***  **Not Missing** | ***n***  **Total** | **%**  **Missing** |
| --- | --- | --- | --- | --- |
| ***Overall Average*** |  |  |  | **0.60%** |
|  |  |  |  |  |
| General Health Status | 513 | 34,208 | 34,721 | 1.48% |
| Mental Health Status | 511 | 34,210 | 34,721 | 1.47% |
| Education | 483 | 34,238 | 34,721 | 1.39% |
| Have a Usual Source of Care | 473 | 34,248 | 34,721 | 1.36% |
|  |  |  |  |  |
| Any Limitation | 167 | 34,554 | 34,721 | 0.48% |
| Arthritis | 153 | 34,568 | 34,721 | 0.44% |
| Angina | 152 | 34,569 | 34,721 | 0.44% |
| Coronary Heart Disease | 149 | 34,572 | 34,721 | 0.43% |
| Other Heart Disease | 145 | 34,576 | 34,721 | 0.42% |
| Myocardial Infarction | 129 | 34,592 | 34,721 | 0.37% |
| Cannot Get Needed Prescription Drugs | 126 | 34,595 | 34,721 | 0.36% |
| Cannot Get Needed Medical Care | 125 | 34,596 | 34,721 | 0.36% |
| High Blood Pressure | 124 | 34,597 | 34,721 | 0.36% |
| Stroke | 121 | 34,600 | 34,721 | 0.35% |
| Emphysema | 113 | 34,608 | 34,721 | 0.33% |
| Diabetes | 104 | 34,617 | 34,721 | 0.30% |
| Asthma | 104 | 34,617 | 34,721 | 0.30% |
| Number of Chronic Conditions | 78 | 34,643 | 34,721 | 0.22% |
| The rest of covariates have no missing data | |  |  |  |

**Supplementary Table 2.** Characteristics of Excluded vs. Included Respondents:

|  | **Excluded** (*n* = 1,802) | **Included**  (*n* = 32,919) | **P value*** |
| --- | --- | --- | --- |
| Any ED Visits, % | **0.270** | 0.190 | 0.000 |
| Any Inpatient Visits, % | **0.270** | 0.160 | 0.000 |
| Number of ED Visits, Mean | **0.440** | 0.270 | 0.000 |
| Number of Inpatient Visits, Mean | **0.480** | 0.230 | 0.000 |
| Total Inpatient Nights, Mean | **4.350** | 1.210 | 0.000 |
| Number of Chronic Conditions, % |  |  |  |
| 0-1 | **0.256** | 0.194 | 0.000 |
| 2-3 | 0.322 | 0.332 |  |
| 4+ | 0.422 | **0.475** |  |
| Had 2+ Chronic Conditions, % | 0.710 | **0.810** | 0.000 |
| **Demographics** |  |  |  |
| Age, Mean | **76.190** | 73.860 | 0.000 |
| Female. % | 0.550 | 0.570 | 0.333 |
| Race/Ethnicity, % |  |  |  |
| White (Non-Hispanic) | 0.660 | **0.796** | 0.000 |
| Black (Non-Hispanic) | **0.158** | 0.082 |  |
| Other (Non-Hispanic) | 0.087 | 0.051 |  |
| Hispanic | 0.096 | 0.071 |  |
| Poverty, % |  |  |  |
| Poor (<100%FPL) | **0.140** | 0.092 | 0.000 |
| Near-Poor (100-125%FPL) | 0.087 | 0.067 |  |
| Low-Income (125-200%FPL) | 0.237 | 0.176 |  |
| Middle-Income (200-400%FPL) | 0.288 | 0.291 |  |
| High-Income (≥400%FPL) | 0.248 | **0.374** |  |
| Interview Not In English, % | 0.050 | 0.040 | 0.008 |
| *Marital Status, %* |  |  |  |
| Single/Never Married | 0.046 | 0.039 | 0.000 |
| Widowed/Divorced/Separated | **0.516** | 0.415 |  |
| Married/Cohabiting | 0.438 | **0.546** |  |
| *Census Region, %* |  |  |  |
| Northeast | 0.207 | 0.192 | 0.174 |
| Midwest | 0.186 | 0.224 |  |
| South | 0.387 | 0.370 |  |
| West | 0.220 | 0.214 |  |
| **Chronic Conditions, %** |  |  |  |
| Any Limitation | **0.710** | 0.580 | 0.000 |
| High Blood Pressure | 0.700 | 0.680 | 0.438 |
| Coronary Heart Disease | 0.220 | 0.190 | 0.173 |
| Angina | 0.100 | 0.080 | 0.178 |
| Myocardial Infarction | **0.160** | 0.120 | 0.001 |
| Other Heart Disease | 0.230 | 0.270 | 0.044 |
| Stroke | **0.170** | 0.120 | 0.000 |
| Diabetes | 0.230 | 0.210 | 0.215 |
| Emphysema | 0.070 | 0.060 | 0.505 |
| Asthma | 0.080 | 0.090 | 0.218 |
| Arthritis | 0.540 | 0.590 | 0.004 |
| **Insurance & Access, %** |  |  |  |
| Had Medicaid | **0.180** | 0.100 | 0.000 |
| Had Private Insurance | 0.380 | **0.490** | 0.000 |
| Cannot Get Needed Medical Care | 0.010 | 0.010 | 0.499 |
| Cannot Get Needed Prescription Drugs | 0.010 | 0.020 | 0.522 |
| P value for Chi-sq test of independence for categorical covariates, and for two-sample t-test for continuous variables. | | | |

**Supplementary Table 3**. Adjusted levels and changes in **emergency department visits** among older adults with multiple chronic conditions (2006-2015)

|  | ***Pre-ACA*** | | | ***Post-ACA*** | | | ***Change*** | |
| --- | --- | --- | --- | --- | --- | --- | --- | --- |
|  | Had ≥1 Visit (%)^(a)^ | Mean Total Visits^(b)^ | | Had ≥1 Visit (%)^(a)^ | Mean Total Visits^(b)^ | | Had ≥1 Visit (pp)^(a)^ | Mean Total Visits^(b)^ |
| *Had* ***…*** *Chronic Conditions* |  |  |  |  |  |  |  |  |
| **0**  (*n* = 2,693) | 10.4‡ [7.9,12.9] | 0.12‡ [0.09,0.16] | | 10.4‡ [5.6,15.2] | 0.15‡ [0.08,0.22] | | 0.0 [-5.6,5.6] | 0.02 [-0.06,0.10] |
| **1**  (*n* = 4,053) | 15.9‡ [13.7,18.1] | 0.20‡ [0.17,0.23] | | 17.0‡ [13.4,20.6] | 0.23‡ [0.17,0.29] | | 1.1 [-3.0,5.1] | 0.03 [-0.03,0.09] |
| **2** (*n* = 5,441) | 14.5‡ [12.7,16.2] | 0.18‡ [0.16,0.21] | | 19.6‡ [16.9,22.4] | 0.30‡ [0.24,0.35] | | 5.2‡ [2.0,8.3] | 0.12‡ [0.06,0.18] |
| **3** (*n* = 5,617) | 17.0‡ [15.1,18.8] | 0.23‡ [0.20,0.25] | | 19.6‡ [16.7,22.6] | 0.31‡ [0.25,0.37] | | 2.7 [-0.8,6.1] | 0.08† [0.02,0.15] |
| **4** (*n* = 4,984) | 17.3‡ [15.5,19.1] | 0.23‡ [0.20,0.26] | | 22.3‡ [19.0,25.7] | 0.34‡ [0.28,0.40] | | 5.1† [1.2,8.9] | 0.11‡ [0.05,0.18] |
| **5+** (*n* = 10,131) | 21.0‡ [19.7,22.3] | 0.30‡ [0.28,0.33] | | 25.2‡ [23.0,27.5] | 0.40‡ [0.35,0.44] | | 4.2‡ [2.0,6.5] | 0.09‡ [0.05,0.14] |
|  |  |  |  |  |  |  |  |  |
| **2+** *(n = 26,173)* | 17.4‡ [16.5,18.3] | 0.23‡ [0.22,0.25] | | 21.7‡ [20.2,23.2] | 0.34‡ [0.31,0.37] | | 4.3‡ [2.5,6.1] | 0.10‡ [0.07,0.13] |
| 95% confidence intervals in brackets. All estimates were adjusted for complex survey design  * p<0.10, † p<0.05, ‡ p<0.01  **^(a)^** Estimates are average marginal probabilities of having at least 1 visit, from logit models fitted to the entire sample (n=32,919)  **^(b)^** Estimates are joint marginal predications from two-part, logit-negative binomial generalized linear models fitted to the entire sample (n=32,919) | | | | | | | | |

**Supplementary Table 4**. Adjusted levels and changes in **inpatient visits** among older adults with multiple chronic conditions (2006-2015)

|  | ***Pre-ACA*** | | | ***Post-ACA*** | | | ***Change*** | |
| --- | --- | --- | --- | --- | --- | --- | --- | --- |
|  | Had ≥1 Visit (%)^(a)^ | Mean Total Visits^(b)^ | | Had ≥1 Visit (%)^(a)^ | Mean Total Visits^(b)^ | | Had ≥1 Visit (%)^(a)^ | Mean Total Visits^(b)^ |
| *Had* ***…*** *Chronic Conditions* |  |  |  |  |  |  |  |  |
| **0**  (*n* = 2,693) | 5.0‡ [3.0,7.0] | 0.07‡ [0.04,0.10] | | 4.6‡ [2.0,7.1] | 0.06‡ [0.02,0.09] | | -0.4 [-3.6,2.7] | -0.01 [-0.06,0.03] |
| **1**  (*n* = 4,053) | 10.4‡ [8.5,12.3] | 0.14‡ [0.12,0.17] | | 9.1‡ [6.4,11.7] | 0.15‡ [0.09,0.21] | | -1.4 [-4.7,2.0] | 0.00 [-0.06,0.07] |
| **2** (*n* = 5,441) | 13.6‡ [11.8,15.3] | 0.18‡ [0.16,0.21] | | 10.3‡ [8.1,12.5] | 0.15‡ [0.11,0.18] | | -3.3† [-6.1,-0.5] | -0.04 [-0.08,0.01] |
| **3** (*n* = 5,617) | 15.3‡ [13.5,17.2] | 0.21‡ [0.18,0.23] | | 13.9‡ [11.6,16.1] | 0.20‡ [0.16,0.23] | | -1.5 [-4.3,1.4] | -0.01 [-0.06,0.03] |
| **4** (*n* = 4,984) | 17.0‡ [15.2,18.8] | 0.24‡ [0.21,0.27] | | 15.6‡ [12.6,18.7] | 0.21‡ [0.17,0.25] | | -1.4 [-5.0,2.3] | -0.03 [-0.08,0.02] |
| **5+** (*n* = 10,131) | 21.3‡ [20.0,22.6] | 0.30‡ [0.28,0.32] | | 21.8‡ [19.7,23.9] | 0.32‡ [0.28,0.36] | | 0.5 [-1.8,2.8] | 0.02 [-0.03,0.06] |
|  |  |  |  |  |  |  |  |  |
| **2+** *(n = 26,173)* | 16.8‡ [15.9,17.6] | 0.23‡ [0.22,0.25] | | 15.4‡ [14.1,16.7] | 0.22‡ [0.20,0.24] | | -1.4* [-2.9,0.2] | -0.01 [-0.04,0.01] |
| 95% confidence intervals in brackets. All estimates were adjusted for complex survey design  * p<0.10, † p<0.05, ‡ p<0.01  **^(a)^** Estimates are average marginal probabilities of having at least 1 visit, from logit models fitted to the entire sample (n=32,919)  **^(b)^** Estimates are joint marginal predications from two-part, logit-negative binomial generalized linear models fitted to the entire sample (n=32,919) | | | | | | | | |

**Supplementary Table 5**. Adjusted levels and changes in **inpatient length of stay** among older adults with multiple chronic conditions (2006-2015)

|  | ***Pre-ACA*** | |  | ***Post-ACA*** | |  | ***Change*** | |
| --- | --- | --- | --- | --- | --- | --- | --- | --- |
|  | Total LOS ^(a)^ | Average LOS ^(a, b)^ | | Total LOS ^(a)^ | Average LOS ^(a, b)^ | Total LOS ^(a)^ | | Average LOS ^(a, b)^ |
| *Had* ***…*** *Chronic Conditions* |  |  |  |  |  |  |  |  |
| **0**  (*n* = 2,693) | 0.42‡ [0.17,0.68] | 0.27‡ [0.14,0.40] | | 0.26‡ [0.07,0.46] | 0.23† [0.04,0.41] | -0.16 [-0.47,0.15] | | -0.05 [-0.27,0.17] |
| **1**  (*n* = 4,053) | 0.87‡ [0.61,1.12] | 0.61‡ [0.43,0.78] | | 0.74‡ [0.41,1.08] | 0.46‡ [0.26,0.66] | -0.12 [-0.52,0.28] | | -0.15 [-0.40,0.11] |
| **2** (*n* = 5,441) | 1.08‡ [0.84,1.31] | 0.73‡ [0.59,0.87] | | 0.83‡ [0.54,1.12] | 0.51‡ [0.36,0.67] | -0.25 [-0.61,0.12] | | -0.22† [-0.43,-0.01] |
| **3** (*n* = 5,617) | 1.21‡ [0.96,1.45] | 0.83‡ [0.67,0.99] | | 0.99‡ [0.71,1.26] | 0.68‡ [0.48,0.88] | -0.22 [-0.60,0.15] | | -0.15 [-0.41,0.11] |
| **4** (*n* = 4,984) | 1.24‡ [1.02,1.45] | 0.84‡ [0.70,0.99] | | 1.15‡ [0.83,1.48] | 0.82‡ [0.55,1.08] | -0.09 [-0.47,0.29] | | -0.03 [-0.33,0.28] |
| **5+** (*n* = 10,131) | 1.50‡ [1.35,1.65] | 0.98‡ [0.89,1.07] | | 1.57‡ [1.33,1.81] | 0.95‡ [0.83,1.07] | 0.08 [-0.20,0.35] | | -0.03 [-0.17,0.11] |
|  |  |  |  |  |  |  |  |  |
| **2+** *(n = 26,173)* | 1.26‡ [1.15,1.36] | 0.85‡ [0.78,0.91] | | 1.14‡ [1.00,1.27] | 0.74‡ [0.65,0.84] | -0.12 [-0.29,0.05] | | -0.11* [-0.23,0.01] |
| ACA: Affordable Care Act; LOS: Length of stay hospitalization nights) | | | | | | | |  |
| 95% confidence intervals in brackets. All estimates were adjusted for complex survey design | | | | | | | | |
| * p<0.10, † p<0.05, ‡ p<0.01 | |  |  |  |  |  |  |  |
| **^(a)^** Estimates are joint marginal predications from two-part, logit-negative binomial generalized linear models fitted to the entire sample (n=32,919) | | | | | | | | |
| **^(b)^** Average length of stay is calculated as the total number of inpatient nights (total LOS) divided by the total number of inpatient visits/discharges | | | | | | | | |

**Supplementary Table 6.** Changes in ED/Inpatient Utilization Around the ACA Estimated in the Main Sample versus in a Fuller Sample Excluding 4 Key Covariates with Missing Data

|  | Had ≥1 ED Visit (%)^(a)^ | | Mean Total ED Visits^(b)^ | | Had ≥1 Inpatient Visit (%)^(a)^ | | Mean Total Inpatient Visits^(b)^ | | Total Length of Stay^(b)^ | | Average Length of Stay^(b,c)^ | |
| --- | --- | --- | --- | --- | --- | --- | --- | --- | --- | --- | --- | --- |
|  | Main Sample | Fuller Sample | Main Sample | Fuller Sample | Main Sample | Fuller Sample | Main Sample | Fuller Sample | Main Sample | Fuller Sample | Main Sample | Fuller Sample |
| *Had …. Chronic Conditions* | Change Pre-Post ACA (Standard Error) | | | | | | | | | | | |
| **0** | 0.03 (2.86) | 0.17 (2.64) | 0.02 (0.04) | 0.02 (0.04) | -0.45 (1.62) | -0.54 (1.43) | -0.01 (0.02) | -0.01 (0.02) | -0.16 (0.16) | -0.11 (0.13) | -0.05 (0.11) | -0.04 (0.09) |
| **1** | 1.09 (2.07) | 1.37 (2.01) | 0.03 (0.03) | 0.04 (0.03) | -1.38 (1.71) | -1.47 (1.63) | 0.00 (0.03) | 0.00 (0.03) | -0.12 (0.20) | -0.10 (0.19) | -0.15 (0.13) | -0.13 (0.12) |
| **2** | 5.18‡ (1.61) | 4.74‡ (1.59) | 0.12‡ (0.03) | 0.10‡ (0.03) | -3.27† (1.42) | -3.65‡ (1.38) | -0.04 (0.02) | -0.04† (0.02) | -0.25 (0.19) | -0.33* (0.19) | -0.22† (0.11) | -0.26† (0.11) |
| **3** | 2.67 (1.77) | 2.29 (1.71) | 0.08† (0.03) | 0.07† (0.03) | -1.47 (1.45) | -2.31 (1.43) | -0.01 (0.02) | -0.03 (0.02) | -0.22 (0.19) | -0.32 (0.21) | -0.15 (0.13) | -0.19 (0.15) |
| **4** | 5.05† (1.97) | 4.57† (1.94) | 0.11‡ (0.03) | 0.11‡ (0.03) | -1.35 (1.86) | -1.65 (1.83) | -0.03 (0.03) | -0.03 (0.03) | -0.09 (0.19) | -0.09 (0.20) | -0.03 (0.16) | -0.03 (0.16) |
| **5+** | 4.24‡ (1.14) | 3.89‡ (1.19) | 0.09‡ (0.02) | 0.09‡ (0.03) | 0.51 (1.18) | -0.11 (1.22) | 0.02 (0.02) | 0.01 (0.02) | 0.08 (0.14) | -0.06 (0.16) | -0.03 (0.07) | -0.09 (0.08) |
|  |  |  |  |  |  |  |  |  |  |  |  |  |
| **2+** | 4.28‡ (0.92) | 3.87‡ (0.92) | 0.10‡ (0.02) | 0.09‡ (0.02) | -1.39* (0.79) | -1.93† (0.77) | -0.01 (0.01) | -0.02* (0.01) | -0.12 (0.09) | -0.20† (0.09) | -0.11* (0.06) | -0.14† (0.06) |
| N | 32,919 | 34,038 | 32,919 | 34,038 | 32,919 | 34,038 | 32,919 | 34,038 | 32,919 | 34,038 | 32,919 | 34,038 |
| * p<0.10, † p<0.05, ‡ p<0.01. All estimates were adjusted for complex survey design  **^(a)^** Estimates are average marginal probabilities of having at least 1 visit, from logit models fitted to each entire sample  **^(b)^** Estimates are joint marginal predications from two-part, logit-negative binomial generalized linear models fitted to each entire sample  **^(c)^** Average length of stay is calculated as the total number of inpatient nights (total LOS) divided by the total number of inpatient visits/discharges  **^(d)^** Analysis with fuller sample excludes 4 key covariates (education, self-rated general and mental health status, and having a usual source of care) which have relatively more missing data (1.5%) but result in the loss of 5% of the eligible sample (n = 34,721). | | | | | | | | | | | | |
